# Pathology and Practice of Medicine

**Published:** 1861-09

**Authors:** Richard J. Dunglison

**Affiliations:** One of the Physicians to the Pennsylvania Institution for the Instruction of the Blind; Honorary Local Secretary to the Sydenham Society of London, etc.


### PATHOLOGY AND PRACTICE OF MEDICINE.

BY RICHARD J. DUNGLISON, M.D.,

ONE OF THE PHYSICIANS TO THE PENNSYLVANIA INSTITUTION FOR THE INSTRUCTION OF THE BLIND; HONORARY LOCAL SECRETARY TO THE SYDENHAM SOCIETY OF LONDON, ETC.

I.—*Researches on Asphyxia; with Observations on the Effects produced by the Hot Bath in Asphyxiated Animals, and its Use in restoring Suspended Animation.* By A. T. H. WATERS, M.R.C.P. Lecturer on Anatomy and Physiology in the Royal Infirmary School of Medicine, etc. (Communicated by Dr. Sharpey.) Proceedings of the Royal Medical and Chirurgical Society, May 14, 1861. (Lancet, May 25, 1861.)

The writer commences his interesting paper by stating that although physiologists are agreed as to the order in which the arrest of the vital actions takes place in asphyxia, they differ as to the duration of the heart's action, and the best mode of treatment in suspended animation. Two important points remain to be decided: first, the period after asphyxia has commenced during which treatment is likely to be successful in restoring animation; and, secondly, the value of the hot bath as a remedial agent. Two questions arise, and experiments on dogs, cats, and rabbits have been instituted with reference to them :—

1. How long does the heart continue to beat in asphyxia?

2. What are the effects of the hot bath on an asphyxiated animal—firstly, after all respiratory movements have ceased, and are not re-excited; secondly, where respiration has been re-excited, and is being feebly carried on?

The following conclusions are drawn by the author from his experiments :—

1. That in asphyxia by submersion, the ventricles of the heart do not, as a rule, cease to contract "in a few minutes after the cessation of the functions of animal life," but that in many instances their action continues for a very considerable period, and that this serves to explain how recovery has taken place after lengthened submersion.

2. That in cases of asphyxia where respiration has altogether stopped, the effects of the hot bath are: to produce an accumulation of blood in the lungs and in the left side of the heart, together with a tendency to coagulate on the part of the blood; that it does not tend to prolong the action of the heart, but rather to paralyze its movements, and diminish the duration of its contractions; that it does not excite respiratory efforts, and prevents artificial respiration being properly carried out.

3. That in cases of asphyxia where respiration has been re-excited and is being feebly carried on, the hot bath, although in some instances it seems to have no immediate bad result, yet has a tendency to produce a fatal issue some hours after its use, by causing extreme congestion of the lungs, together with consolidation and collapse of the pulmonary tissue.

The following practical inferences are drawn from the above conclusions:—

1. That efforts to restore animation should be made in all cases where asphyxia has not been of very prolonged duration.

2. That the prolonged use of the hot bath in asphyxia is not only inefficacious, but dangerous; and that its temporary use appears to be attended by no direct benefit. So far as any means similar to that of the hot bath are likely to produce respiratory movements, the alternate dashing of hot and cold water on the body is probably the most efficacious.

3. That it appears safer practice to omit all artificial treatment, when respiration is going on feebly, than to make use of the hot bath.

4. That in the treatment of asphyxia all efforts should be primarily directed to restoring, or continuing, the respiratory movements; and all measures tending to load the lungs or embarrass the respiration should be avoided.

Dr. Waters's recommendations are, the use of friction, moderate temperature, and the method of Dr. Marshall Hall. By this method, Dr. Sylvester had obtained a displacement of one cubic inch only, while in his (Dr. Waters's) experiments on the dead body he had succeeded in displacing eight or ten cubic inches, which would probably be sufficient. He believed that the position in which the body was placed in Dr. Marshall Hall's method was of great importance in allowing fluids to fall out of the mouth.

Dr. Babington was not sure whether experiments upon dogs respecting warm baths were applicable to human beings; the warm bath would probably be more injurious to asphyxiated dogs, from the fact that the skin of the dog was remarkably thick, and it was known that he did not perspire. Dr. Chambers said that asphyxia in new-born children was not removed by the use of the warm bath.

At a subsequent meeting of the society, a letter was read from Sir B. Brodie, in which the writer stated that he had never, in his experiments on this subject, known the rhythmical contractions of the heart to continue for more than four minutes and a half after complete submersion; and believed if they had once ceased in asphyxia they could not be restored. Cases of recovery after a longer submersion he attributed to the action of the heart having previously ceased in a state of syncope.

(For an interesting discussion upon the same subject, see an analysis of a paper by Dr. Christian in favor of Dr. Sylvester's method, North Amer. Med.-Chir. Review, May, 1861.)

II.—*The Liverpool Fever at Alexandria.* (Medical Times and Gazette, June 1, 1861, p. 588.) By J. F. OGILVIE, M.D., Surgeon to H. B. M. Consulate, etc. (See also N. A. Med.-Chir. Review, July, 1861.)

In the last number of this journal, we called attention to a visit of exanthematic typhus fever to Liverpool, dating from the arrival at that seaport of a number of uncleanly Egyptian sailors. The same crew, transhipped into another vessel, touched at Malta on their return to Alexandria, and left behind them a fever of a malignant type, of which several persons died. On the arrival of the steamer in Alexandria, Dr. Ogilvie tells us, some twenty-eight or thereabout of the crew were admitted into the Native Hospital for rheumatism, meningitis, dysentery, and other non-contagious diseases, but not one with typhus or other contagious fever, of whom five or six have since died. Of seven Englishmen on board, six were affected at the time of landing, or immediately after, with fever, but in different degrees of intensity. In all of the cases, the type of the fever was rather inflammatory than typhoid, and in all these was a degree of fidgety, restless excitement and affection of the nervous system, quite out of proportion to the other symptoms. In only one case was there any symptom leading to a suspicion of a contagious type of fever. All the Englishmen made good recoveries up to the forty-second day after their arrival at Alexandria. No case of sickness, either in the hospitals or in the town, was traceable to the crew of this unfortunate steamer.

III.—*Some Effects of Reflex Nervous Irritation produced by Syphilitic Disease of the Bones of the Skull.* A Paper by MR. HENRY LEE, read before the Royal Medical and Chirurgical Society, May 28, 1861. (Medical Times and Gazette, June 15, 1861.)

This paper seems to be supplementary to one presented at a previous session by the same author. In one of the cases detailed at that time, deafness and impaired voluntary power coexisted with extensive ulceration; in a second, there were long-continued spasms of some of the muscles of the face and body; and in a third, delirium and coma were present. The author remarks that a state of chronic spasm may be produced in blood-vessels as well as in muscles by nervous irritation; and the post-mortem examination in two cases cited showed the irritation to have originated in the bones of the skull at a distance from the origin of any of the motor nerves, and he therefore concludes that as the parts at the base of the brain were healthy, the venous irritation depended upon reflex action. The same action which would produce spasm of the muscles at one time might produce spasm of the blood-vessels at another, and thus interfere with the process of nutrition. To this cause the author attributed the ulcerations. In two of the cases examined post-mortem, the inner table of the skull had been extensively diseased, and had been the cause of disease of the dura mater. In both cases, the parts at the base of the brain were entirely free from disease; and it was particularly noticed that in those situations where the internal table had been removed (in one case artificially) the membranes of the brain were healthy; but in those parts where the diseased bones remained, that the membranes were also diseased. The conclusion drawn by the author was that where portions of bone were exposed by disease, the external and middle tables might be removed by the trephine without any danger; and that where the internal table was diseased, and the locality of that disease was indicated, which it might be, and the exposed external table, then the whole thickness of the skull might be removed by the trephine.

In a case detailed, characterized by periodical attacks of pain in the head, staggering, etc., the author remarks that there was, during the occurrence of the attacks, an affection of the muscles of one particular limb and of the blood-vessels of the same part, as indicated by the bloodless condition of the skin. It was doubtless, like the other cases, an instance in which irritation of the dura mater by disease of the bone had, by reflex action, produced the symptoms which had been described.

IV.—*The Diseases of the Kidneys, accompanied by Albuminuria.* By DR. W. H. DICKINSON. (Medical Times and Gazette, June 8, 1861.)

In a previous essay on the subject, the author maintained, on anatomical grounds, that the disease which occasions the smooth mottled kidney is essentially different from that which gives a granular surface to the organ. The first is characterized by excess of cell-growth within the tubes, and is, in fact, chronic nephritis; while the other is the result of a degeneration commencing in the intertubular structures. The purpose of the present paper is to add the clinical details. These were obtained, in part, from an analysis of 369 well-marked fatal cases of renal disease extracted from the records of St. George's Hospital during a period of ten years, while other particulars were obtained from a more minute examination of cases under the author's notice during life, taking care to use only those in whom the state of the kidney had been attested by post-mortem examination.

We shall endeavor to make our own classification of symptoms, etc., diagnostic of each, from the numerous details furnished in Dr. Dickinson's excellent paper.

| TUBULAR DISEASE, (Chronic Nephritis.) | GRANULAR DEGENERATION. |
|---|---|
| *Sex.*—More frequent in the male than the female, as 3 to 1. | More frequent in the male, as 2 to 1. |
| *Age.*—In early life; attaining its greatest frequency between ages of 20 and 30. | Never seen except in adults; most common after the age of 40. |
| *Causes.*—Various causes of renal hyperæmia; exposure to cold, elimination of the poisons of scarlatina and other exanthemata; the irritation of turpentine or cantharides given medi- | Can scarcely ever be attributed to any external cause. Is the result of gradual changes originating in the intertubular tissues of the organ, and depending upon some constitutional |

cinally; the vicarious secretion of bile, or the congestion of successive pregnancies.

*Duration.*—Within 6 months; 26 out of 34 fatal cases under the author's observation terminated within this period, while only 2 survived the year.

*Symptoms.*—Albuminuria; dropsy of the cellular tissue, pleuræ, peritoneum, and pericardium, (and frequently in the order mentioned;) diarrhœa, especially in the later stages; pain in the loins; bloated aspect and ivory pallor.

*Head Symptoms.*—Epileptic convulsions occur occasionally, sometimes followed by coma, especially in those who have been reduced by vomiting or diarrhœa.

*Condition of the Urine.*—The urine is scanty, except during the later stages, or while a temporary attack is passing away. It is usually acid, and often deposits uric acid and urate of soda. It is discolored with blood in a large proportion of cases, especially at the outset, and yields a bulky precipitate of albumen, independently of any such admixture. The specific gravity ranges from 1010 to 1030; but on the whole does not depart much from the limits of health.

*Microscopic Characters.*—Renal epithelium is deposited in a natural state, or containing oil-globules, or converted into pus-cells. The fibrinous cylinders are to be regarded as casts only of the vice which gains ascendency with advancing years. Those predisposed to gout and tubercle are especially liable.

Impossible to fix the duration, because of the indefinite nature of its accession; period may vary from 4 weeks to 10 years.

Gradual change from health to infirmity for months, perhaps, as shown by slight fullness of the ankles at first; sharp features; sallow complexion; copious urine, passed with frequency, especially at night. In the later periods, dropsical effusion only sometimes comes on when the urine is no longer profuse; with withered aspect, sharpened features, and a muddy skin.

Either epileptic convulsions, or, what is in some measure characteristic, patients often pass gradually into a comatose state without any such warning, the change being preceded by dimness of sight, giddiness, peculiarity of manner, or delirium. Extravasation of blood within the cranium is peculiarly apt to take place in connection with granular disease; the latter was present in nearly one-half of the cases of apoplexy subjected to examination in St. George's Hospital.

The urine is increased in quantity, except in the later periods, and is usually paler than natural, being comparatively seldom discolored with blood. The albumen is often in minute quantity, and the specific gravity low, varying from 1008 to 1020.

The casts are various in character, but those most often present are of a coarse, granular texture, and may be regarded as very characteristic of the disease.

straight tubes; they are of four varieties, viz.: transparent, uniform glassy cylinders; similar cylinders imbedding entire epithelial cells; casts of granular appearance, containing epithelium in a state of disintegration; and, lastly, such as contain blood-corpuscles. The casts imbedding entire epithelial cells are the most characteristic.

*Intercurrent Diseases.*—Inflammation of the serous membranes and of the lungs occur frequently.

Bronchitis is of frequent occurrence. Valvular disease of the heart occurs more frequently than can be attributed to accidental coincidence.

The presentation of so interesting and valuable a paper to the Royal Medical and Chirurgical Society elicited remarks by Dr. C. J. B. Williams and others. The former, while commending Dr. Dickinson's researches, stated his views to be, that the anatomical and other peculiarities were different stages of one disease, rather than separate and definite affections. Dr. Gull thought the author should have referred also to a third form of kidney disease, depending on amyloid degeneration of the arteries, associated with scrofula and phthisis; but it was evidently not the intention of Dr. Dickinson, as he himself stated, to give a complete history of the various affections often grouped together as Bright's disease, but to distinguish only the two forms alluded to in his paper.

V.—*Cases of Disease of the Pons Varolii.* By HERMANN WEBER, M.D. (Lancet, June 1, 1861.)

The three cases detailed by Dr. Weber are of great interest, on account of the rarity of the affection. Two of the cases are of especial interest, on account of the isolated and limited lesion, and the comparatively slow progress of the disease allowing time for repeated observations. The third case corroborates some of the inferences deduced in regard to the others, though it really possesses so little else that is interesting in its details, that we have limited our remarks to the other two cases.

In Case 1, affected with tuberculosis of the lungs, the symptoms were at first vertigo and weakness of the limbs of the right side, without alteration of sensibility; three months afterward increased paralysis of movement in the right arm and leg, with imperfect anæsthesia in the left side of the face and tongue, and contraction of the pupils, especially the right; followed in the next month by contraction and rigidity and diminution of sensibility in the limbs of the right side. These symptoms were followed by weakness of those of the left side, and defective mastication, deglutition, and articulation; afterward by vertigo and convulsions, momentary loss of consciousness, but permanent loss of articulation, deglutition, and protrusion of tongue. The paralysis of the limbs of the right side was complete, of the left almost so; there was anæsthesia in those of the right, distinct diminution of feeling in those of the left side. Reflex function of the limbs was not quite destroyed; both pupils were contracted; death occurred under the phenomena of paralysis of respiration.

In Case 2, affected with chronic hydrocephalus and tuberculosis of the lungs, we find symptoms very analogous to those of Case 1. In comparing the symptoms of the two cases during the first period, when the anatomical alterations were almost the same in both, we find, as principal differences, that in Case 2 the limbs of the side opposite the central lesion were attacked by fits of convulsions previously to their being paralyzed; that further, in the same case, pain in the side of the face corresponding to the lesion preceded the anæsthesia; and that the same child had later fits of general shaking—phenomena which were absent in Case 1. These phenomena of one case only may not be due necessarily to the palpable local affection.

Many symptoms correspond in the two cases, viz.: motor affection, first of the arm, then also of the leg of the side opposite to the anatomical lesion; attacks of vertigo; contraction of the pupils, more so of the one opposite to the tumor; sensitive affection of the face and tongue on the side of the tumor, (due to the relation of the latter to the fifth nerve;) the sensibility of the limbs of either side unaffected, until to the second period, when, in Case 1, anæsthesia came on in those of the side opposite to the lesion; wasting of the paralyzed limbs, etc., and integrity of the intellectual functions and special senses throughout.

In the post-mortem examination of Case 1, a tubercle of the diameter of half an inch was found in the substance of the left half of the pons Varolii, in close proximity to the superficial origin of the left quintus, with softening of the surrounding tissue, extending posteriorly almost to the floor of the fourth ventricle, and toward the right side beyond the middle line. There was hemorrhage into the softened tissue, and the left trigeminus was thinner than the right. In Case 2, in addition to the phenomena of tubercles of the lungs and tubercular meningitis, with increased amount of fluid in the dilated ventricles, a tubercle larger than a pea was found in the lower portion of the right half of the pons, near the right quintus, with atrophy of the latter. The nerve-tissue of the pons surrounding the tubercle was softened, of a yellowish-red color, exhibiting under the microscope many granule corpuscles, (exudation corpuscles.)

The physiological inferences which offer themselves are, according to Dr. Weber:—

1. That the nerve-fibres for the limbs, passing through the pons, as well motor as sensitive, decussate below the pons.

2. That there are no sensitive fibres for the limbs in the anterior (inferior) portion of the pons.

3. That the posterior (superior) portion of the pons contains sensitive fibres for the limbs.

4. That the intellectual functions of the brain, and also of the special senses, are independent of the pons.

5. That nerves regulating the state of the pupils are in close connection with the pons.

6. That extensive lesion of the pons is associated with disturbance of deglutition, articulation, and respiration.

VI.—*On Sour-smelling Perspiration in Acute Rheumatism, and its Significance as a Symptom.* By THOMAS INMAN, M.D., Physician to the Royal Infirmary, Liverpool. (London Medical Review, June, 1861, p. 582.)

Theories respecting acute rheumatism have been built on copious sour-smelling perspiration as a symptom of this disease, and distinct lines of practice founded upon them. For instance, it has been maintained, as Dr. Inman remarks, that sweating is an effort of nature to eliminate some poison from the blood; while another view is, that as the sour odor is due to lactic acid, so that acid must, in some way, be concerned with the poison to be eliminated. We therefore find medical men, who adopt these theories, encouraging perspiration, giving daily purges, and acting freely on the kidneys with their diuretics; or else they administer alkalies to neutralize the acid in the blood.

The writer asks the following questions, and answers them himself:—

1. *Is it a fact that all cases of acute rheumatism are attended with a sour-smelling secretion from the skin?* Perspiration is absent in about one-third of the cases which have come under his observation, and has been excessive only in one-fourth. A sour odor is not perceptible unless the sweating is very considerable.

2. *Does the occurrence of profuse sour sweat relieve the other symptoms;* and

3. *Are those cases the mildest in which the perspiration is the freest and sourest?* Dr. Inman has never yet seen the symptoms relieved by the occurrence of perspiration, or by its excess. After an experience of over twenty years, he has arrived at the conclusion that the patient improves as the skin becomes dry; that a return of perspiration is always accompanied with an aggravation of the other symptoms, and that those cases are the worst in which the sweating is most excessive in quantity and most acid in odor.

4. *Is the sweat sour when first it is produced, or is its odor the result of decomposition?* If we can ascertain—1, that the perspiration when it first appears is free from any unusual odor; 2, that no sour smell is noticed after a complete change of body linen and sheets, and for a considerable period subsequently, we have *prima facie* proof that it is not the natural perspiration alone that gives rise to the odor.

5. *Does a similar sour odor accompany perspiration in other diseases, and, if so, have they anything in common with acute rheumatism?* Various diseases, incidentally attended with sour sweating, are cited by the author; but they have little in common except great poverty of the blood as regards globules, richness in fibrin, and constitutional debility.

6. *Why is the perspiration of a rheumatic patient more frequently sour than that of other people?* It is for this reason, that when the sweating is abundant, the pain is always so severe that the patients cannot endure the motion consequent upon an attempt to change their body linen, sheets, etc. There is every reason to believe that the sour smell is the result of decomposition *after* the fluid has been secreted, and deposited outside the body; consequently no theory can fairly be founded upon the change as regards a poison being eliminated, that poison being lactic acid.

7. *Are the results of the eliminant or alkaline plans of treatment so conspicu-*

*ously successful as to warrant us in taking them as corroborative of the theories upon which they are founded?* Dr. Inman has never satisfied himself that the sour smell has diminished under the use of alkalies so rapidly as it does when lime-juice alone is employed. But all conclusions as to results of medical treatment were vitiated as soon as it was ascertained that in some few instances the whole symptoms of acute rheumatism would subside rapidly without any special treatment whatever. On the other hand, it is true, that the majority of cases evince no tendency to spontaneous cure, that lime-juice alone does not always cure rapidly, and that patients do get well when taking diaphoretics, purgatives, and alkalies. Time and average success are adduced in favor of the lime-juice treatment.

VII.—*The Value and the Fallacy of Statistics in the Observation of Disease.* By David W. Cheever, M.D. (Boylston Prize Essay, Boston, 1861.)

An immense number of reasons why statistics should be regarded as unreliable and scarcely ever worthy of being appealed to, can be readily collected together by one who, like Dr. Cheever, makes their collection a specialty. But they have their value in a limited degree: and far greater in some departments than in others, as the writer himself states. This value descends in accuracy by a progressive ratio, and in the following order: mortality and births; hygiene; etiology; pathology; therapeutics. We find the statistics reliable in proportion to the simplicity of the *data* from which they are calculated.

The author refers to facts established by the numerical method, such as the conclusions that tubercles in any organ of the body, after the age of fifteen years, involve their presence in the lungs; that chronic peritonitis, too, indicates pulmonary tubercles; that phthisis so often commences in the upper lobes, that we have been led to call those cases where the indications of its presence are found first at the base of the lungs, the *anomalous* development of tubercles, etc.; points on which no theory and no speculation could ever have led to these results of numeration and averages.

Since the use of a very large number of observations, however, in every case is impracticable, how shall we know what value to attach to statistical conclusions derived from a limited series of facts only? By the calculation of probabilities, which must be received as demonstrated authority by those who do not choose to study it mathematically. But this method proves to us that the probability of a given event happening does not exactly coincide with the actual number of times it has been observed to happen, but varies between limits somewhat greater and somewhat less than the number observed; and that these limits, moreover, are wider in proportion as the observations are few, and approach nearer as the observations become more numerous.

Let us suppose, for example, as mentioned by Dr. Bartlett, that 500 cases of a given disease have been subjected to a given treatment, with the result of 100 deaths, and 400 recoveries; and another set of 500 cases of the same disease have been subjected to a different treatment, with the result of 130 deaths and 370 recoveries. In the first class, the ratio of mortality is as 20,000 to 100,000; in the second class this ratio is as 26,000 to 100,000; the difference between the two being 6000 in 100,000. An application to these numbers of the law of probabilities, as deduced by the mathematical formula of M. Gavarret, shows that the limit of possible variation is equal to 7508 in 100,000.* We cannot reasonably conclude, therefore, that the first method of treatment is better than the second, because the difference in the result *falls below* the limit of possible variation by the calculation of probabilities; a variation which may be the effect of chance.

But by extending our observations to twice the number of similar cases in which the ratio of mortality remains in each class the same, we find the following results: the limit of possible variation, ascertained by the calculation of probabilities, when applied to 1000 cases, instead of 500, sinks from 7508 in 100,000 to 5306 in 100,000; which is *surpassed* by the observed difference in the ratio of mortality, this being as 6000 in 100,000. Here then we have a positive demonstration of the superiority of the first mode of treatment over the second; and this demonstration obtained solely by increasing the number of our observations.

In all statistical calculations, each series of relationships and phenomena must be fixed enough to be comparable; must consist of large numbers; the limits of variability must be determined by the calculation of probabilities; and the series of possible causes must remain uniform. Dr. Cheever has almost exhausted the discussion of the value of the numerical method of observation.

VIII.—*A Statistical Account of* 476 *Cases of Acute Rheumatism admitted into the Wards of the Middlesex Hospital during the Years* 1853 to 1859 inclusive. By GEORGE WILLIAM FLEETWOOD BURY, Esq., F.R.C.S., etc. (Brit. and For. Medico-Chirurg. Rev., July, 1861.)

From a large group of cases, such as are embraced in the tables of Mr. Bury, certain practical deductions may be made, having reference to the points of interest connected with the disease alluded to. Thus, we learn the influence of sex, age, hereditary predisposition, etc.

*Complications.*—Omitting entirely, as complications, any example of simply old heart disease—the result of previous rheumatic attacks—and including only those instances of cardiac mischief which were considered either to have originated with the present rheumatic attack, or those in which, during the present attack, new cardiac disease had been grafted on old structural and rheumatic changes, we find that 253 of the cases, or about 53·7 per cent. of those attacked with acute rheumatism, suffered from heart complication of one kind or another. Women suffered in a much greater proportion than men from such complications; in the latter, the percentage was 45·3; in the former, 62·2.

Endocarditis was four times as frequent a complication as pericarditis, and twice as frequent as endo-pericarditis.

*Hereditary tendency.*—In 67 cases, or rather more than 14 per cent., relatives were subject to rheumatism, the predisposition seeming to be much more frequently inherited from the father than from the mother, and its probable derivation from both being of comparatively rare occurrence.

*Age.*—From the table of relative frequency of the affection, and its heart complications, at various ages, we deduce the following conclusions: Acute

* Philosophy of Medical Science.

rheumatism is a disease of the period of life embraced between 15 and 30, occurring especially during the first quinquennial interval, and then diminishing as age advances. The heart complications, in the cases observed by Mr. Bury, prevailed at irregular periods, as is exhibited in the following order of frequency: 15 to 20 years of age, under 10, between 10 and 15, 20 and 25, 30 and 35.

*Joints affected.*—Distal joints of a limb are more liable to be first attacked, and a tendency exists, so great as almost to amount to a rule, for the disease to commence in the lower extremity and travel upward with a certain regularity. The joints of the lower extremity alone were affected exactly twice as often as those of the upper extremities alone. The erratic and diffusive tendency of the disease is shown in the fact that the joints of both extremities have been attacked more than twice as often as all the other cases put together.

*Duration of attack.*—The average length of stay in the hospital—49 days—may, for several plausible reasons stated, be considered as fairly representing the duration of the attack.

*Period of occurrence of heart complications*—(their advent dating from the first day on which any distinctly abnormal sound was heard.) They may occur as early as the second day of the disease, and during the first thirteen days the liability is at a maximum. The fifth and eighth days are those of greatest frequency of complication.

In endo-pericarditis, endocarditis is said by Mr. Bury generally to precede pericarditis by a day or two at the least, though we confess to an admiration and doubt of such nice diagnostic skill as can decide upon the non-existence of either one form or the other of cardiac inflammation but a few hours before the appearance of both conjoined.

*Previous attack.*—Nearly one-half the cases had suffered previously; about 66 per cent. of these but once.

*Period of the year during which acute rheumatism is most prevalent.*—June and October, between which there are so few points of analogy, are, inexplicably enough, the months of greatest frequency.

